# Development and temporal validation of a prediction model for live birth in infertile women with polyendocrine metabolic ovarian syndrome: a retrospective cohort study

**DOI:** 10.3389/frph.2026.1891181

**Published:** 2026-07-28

**Authors:** Peng Liu, Shuhong Wang, Yubo Lu, Hanbing Jia, Yue Geng, Cuijuan Cao, Le Han, Yingjie Zhou

**Affiliations:** 1 Department of Clinical Laboratory, Hebei Reproductive Health Hospital, Shijiazhuang, Hebei, China; 2 School of Electronic Information and Electrical Engineering, Shanghai Jiao Tong University, Shanghai, China

**Keywords:** infertility, live birth, nomogram, polycystic ovary syndrome, polyendocrine metabolic ovarian syndrome, prediction model, temporal validation

## Abstract

**Objective:**

To develop and temporally validate a prediction model for delivery of a live-born infant within 365 days after the initial infertility consultation in infertile women with a documented diagnosis of polycystic ovary syndrome (PCOS), which was renamed in 2026 as polyendocrine metabolic ovarian syndrome (PMOS), using routine clinical and basal endocrine variables.

**Methods:**

This single-center retrospective cohort study included infertile women with a documented diagnosis of PCOS and an index date between December 1, 2021, and December 31, 2024. The index date was the first infertility consultation at which baseline clinical assessment and basal endocrine testing were completed. Patients were assigned to the development cohort (December 1, 2021–December 31, 2023) or the same-center temporal validation cohort (January 1–December 31, 2024). Outcome ascertainment ended on December 31, 2025. Multivariable logistic regression was used to predict delivery of a live-born infant within 365 days under routine individualized care.

**Results:**

Among 610 women, 389 were in the development cohort and 221 in the temporal validation cohort. Live-birth proportions within 365 days were 44.7% and 44.3%, respectively. Complete-case model development and validation included 349 and 203 women, respectively. The final model included age, body mass index, infertility duration, gravidity, luteinizing hormone/follicle-stimulating hormone ratio, tubal/pelvic factors, and uterine factors. Older age (OR = 0.91, 95% CI: 0.85–0.98), higher body mass index (OR = 0.92, 95% CI: 0.86–0.98), longer infertility duration (OR = 0.85, 95% CI: 0.77–0.94), higher luteinizing hormone/follicle-stimulating hormone ratio (OR = 0.71, 95% CI: 0.55–0.91), and uterine factors (OR = 0.37, 95% CI: 0.15–0.80) were associated with lower live-birth probability. The AUCs were 0.735 and 0.738, and the Brier scores were 0.208 and 0.205, respectively.

**Conclusion:**

This model showed moderate discrimination and acceptable calibration for predicting delivery of a live-born infant within 365 days after initial infertility consultation. Multicenter external validation and impact studies are needed before broader clinical application.

## 1 Introduction

Polycystic ovary syndrome (PCOS), renamed in 2026 as polyendocrine metabolic ovarian syndrome (PMOS) through a global consensus process and announced by the Endocrine Society, is one of the most common endocrine and metabolic disorders among women of reproductive age, with a global prevalence of approximately 5%–15% (1, 2). It accounts for up to 70%–80% of cases of anovulatory infertility (3–5). Patients with PCOS commonly present with menstrual irregularities, chronic anovulation, hyperandrogenism, and polycystic ovarian morphology, and may also have metabolic abnormalities such as obesity, insulin resistance, and impaired glucose metabolism (6). Owing to the marked heterogeneity of clinical phenotypes, patients differ substantially in ovulatory function, endocrine status, endometrial environment, and coexisting infertility-related factors. Consequently, pregnancy and live birth outcomes vary considerably among individuals. Accurate assessment of reproductive prognosis before treatment therefore remains an important issue in clinical practice (7–9).

Clinical management of infertility in women with PCOS mainly includes lifestyle intervention, weight management, ovulation induction, and assisted reproductive technology when necessary (10). However, treatment responses and final reproductive outcomes are not consistent across patients. Some patients achieve live birth after conventional ovulation induction, whereas others fail to achieve a successful outcome despite multiple treatment cycles (11). Traditional assessment often relies on empirical judgment based on individual indicators, such as age, body mass index (BMI), duration of infertility, and basal sex hormone levels, which may not fully capture the combined effects of multiple factors on reproductive outcomes. Developing individualized prediction models by integrating multidimensional clinical variables may improve the accuracy of prognostic assessment and support preliminary prognostic counseling and early risk stratification at the initial infertility assessment.

Live birth is a more clinically meaningful endpoint than biochemical pregnancy or clinical pregnancy, as it more comprehensively reflects the overall reproductive benefit from conception to delivery and better aligns with the primary concerns of both patients and clinicians (12). In recent years, several studies have attempted to develop prediction models for pregnancy or live birth outcomes in patients with PCOS. However, existing models have mainly focused on specific treatment strategies or populations undergoing assisted reproduction. Some models depend on treatment-related parameters or specialized laboratory indicators, such as gonadotropin dosage, number of oocytes retrieved, and anti-Müllerian hormone (AMH), which limits their direct applicability at the initial consultation or in routine outpatient settings (13). In addition, most models have undergone only internal validation or random split-sample validation, whereas temporal validation using patients from a later period is lacking; therefore, their stability remains to be further evaluated (14). In contrast, female age, BMI, duration of infertility, reproductive history, luteinizing hormone/follicle-stimulating hormone (LH/FSH) ratio, and tubal/pelvic and uterine factors are routinely available at the initial assessment. A prediction model based on these variables may provide individualized risk stratification at the early stage of patient evaluation and has favorable potential for clinical implementation (15).

Therefore, based on a single-center retrospective cohort of infertile women with PCOS, this study used live birth as the primary outcome and developed a multivariable logistic regression prediction model using routine clinical characteristics and basic endocrine indicators. A nomogram was then constructed, and model performance was evaluated using a temporal validation cohort. This study aimed to establish an easily applicable prediction tool based on readily available variables to estimate the probability of live birth in infertile women with PCOS, thereby providing a tool for preliminary individualized counseling and early risk stratification at the initial infertility assessment.

## 2 Materials and methods

### 2.1 Study design and participants

This was a single-center retrospective cohort study that included infertile women with a documented clinical diagnosis of polycystic ovary syndrome (PCOS) at our center and available follow-up data on pregnancy outcomes. Demographic characteristics, infertility-related information, reproductive history, basal endocrine indicators, diagnostic information, and pregnancy outcome data were collected.

#### 2.1.1 Definition of the study population

The study population comprised infertile women with a documented clinical diagnosis of PCOS in the electronic medical record during routine care at our center. Because this was a retrospective study based on routinely collected clinical data, a standardized retrospective reassessment of all individual diagnostic components of PCOS was not feasible for every participant. Detailed information on menstrual history, clinical and biochemical hyperandrogenism, ultrasonographic morphology, and exclusion testing for alternative endocrine disorders was not uniformly available for retrospective verification. Because this retrospective study included women diagnosed between 2021 and 2024, case identification was based on the clinical diagnosis and diagnostic criteria routinely used during that period, which employed the term polycystic ovary syndrome (PCOS), before the 2026 renaming of the condition as polyendocrine metabolic ovarian syndrome (PMOS). The terms PCOS and PMOS refer to the clinical syndrome and should not be confused with polycystic ovarian morphology (PCOM), which represents only one imaging feature that may contribute to diagnosis. PCOS phenotypic classification (e.g., Rotterdam phenotypes A–D) was not routinely recorded in the electronic medical records during the study period and therefore could not be evaluated in the present retrospective analysis.

Accordingly, the study population should be interpreted as women with infertility and a recorded clinical diagnosis of PCOS, rather than women in whom PCOS was confirmed as the sole cause of infertility. Women with coexisting tubal/pelvic factors, uterine factors, male factors, thyroid disease, or abnormal glucose metabolism were not excluded because the model was designed to estimate the probability of delivery of a live-born infant within 365 days after the index consultation in a real-world infertility cohort.

The inclusion criteria were as follows: (1) infertility with a documented clinical diagnosis of PCOS in the medical record; (2) clearly documented registration date; and (3) availability of baseline clinical data and follow-up information on pregnancy outcomes. The exclusion criteria were as follows: (1) missing primary outcome information or incomplete outcome ascertainment through day 365; (2) obvious data-entry errors that could not be verified; and (3) duplicate records.

#### 2.1.2 Participant flow and cohort formation

A total of 746 records were initially screened. Records were excluded if they did not meet the PCOS/infertility inclusion criteria (*n* = 18), lacked adequate baseline clinical assessment or basal endocrine testing (*n* = 26), had missing or incomplete 365-day outcome ascertainment (*n* = 72), were duplicate records (*n* = 11), or contained unverifiable data-entry errors (*n* = 9). After these exclusions, 610 women remained in the eligible analytic cohort.

### 2.2 Variables and outcome definitions

The collected variables included female age, body mass index (BMI), type of infertility, duration of infertility, gravidity, parity, number of miscarriages, number of ectopic pregnancies, basal endocrine indicators, and diagnosis-related factors. Basal endocrine indicators included follicle-stimulating hormone (FSH), luteinizing hormone (LH), estradiol (E2), progesterone (P), prolactin (PRL), and testosterone (T), and the LH/FSH ratio was calculated. Diagnosis-related factors included tubal/pelvic factors, ovulatory dysfunction, uterine factors, male factors, thyroid disease, and abnormal glucose metabolism.

#### 2.2.1 Basal endocrine assessment

Basal endocrine blood samples were collected during routine clinical care on menstrual cycle day 2 or day 3 in women with spontaneous menstrual bleeding. For women with amenorrhea or markedly irregular cycles, blood sampling was performed after exclusion of pregnancy and, when clinically indicated, on day 2 or day 3 after progestin-induced withdrawal bleeding according to the routine protocol at our center. Amenorrhea itself was not used as an exclusion criterion, because women with PCOS-related menstrual dysfunction could still receive individualized infertility treatment and complete outcome follow-up. Serum follicle-stimulating hormone (FSH), luteinizing hormone (LH), estradiol (E2), progesterone (P), prolactin (PRL), and testosterone (T) were measured in the hospital clinical laboratory using a chemiluminescence immunoassay (CLIA) on a Beckman Coulter DxI 800 immunoassay analyzer. The reporting units were mIU/mL for FSH and LH, pg/mL for E2, and ng/mL for P, PRL, and T. The LH/FSH ratio was calculated by dividing the measured LH concentration by the measured FSH concentration. Laboratory quality control procedures and reference intervals followed the standards of the hospital clinical laboratory. Information on recent exposure to hormonal contraceptives, progestins, ovulation-induction agents, insulin-sensitizing therapy, or other medications that could influence basal endocrine measurements was not uniformly available in the retrospective records. Information on standardized medication washout before testing was also not consistently documented. Therefore, no uniform washout criterion could be applied, and residual effects of prior medication exposure on basal endocrine measurements cannot be excluded.

#### 2.2.2 Definition of diagnosis-related factors

Ovulatory dysfunction was coded as present when oligo-ovulation, anovulation, or another ovulatory disorder had been documented by a reproductive specialist during routine infertility assessment. In routine clinical practice, this assessment was based on menstrual history together with available clinical, ultrasonographic, and/or hormonal information. Because detailed cycle-level ovulation monitoring and uniform biochemical confirmation were not consistently available in the retrospective records, a standardized retrospective threshold was not applied.

Male-factor infertility was coded as present only when semen-analysis abnormalities had been documented by the treating reproductive specialist as clinically relevant male-factor infertility. A uniform retrospective semen-parameter threshold was not applied because detailed semen parameter values and interpretive reports were not consistently available for all couples. Mild semen abnormalities and combined male and female infertility factors were not consistently recorded as separate male-factor diagnoses and may therefore have been undercaptured. Accordingly, the low observed prevalence of male-factor infertility should be interpreted cautiously and should not be interpreted as indicating that male evaluation was absent.

The diagnosis-related variables were extracted from routine medical-record documentation and were used as baseline prognostic characteristics rather than as standardized etiologic classifications.

#### 2.2.3 Handling of missing data

Missingness was assessed separately for all candidate predictors and the outcome in the development and temporal validation cohorts. The eligible analytic cohort was retained for descriptive analyses after application of the prespecified eligibility criteria and data-quality checks.

For final model estimation and temporal validation, complete-case analysis was performed using the seven final predictors: female age, body mass index, infertility duration, gravidity, LH/FSH ratio, tubal/pelvic factors, and uterine factors. The LH/FSH ratio was considered missing when either LH or FSH was unavailable. No imputation was performed.

In the development cohort, 40 of 389 women had missing data for at least one final predictor, leaving 349 complete cases for model development. In the temporal validation cohort, 18 of 221 women had missing data for at least one final predictor, leaving 203 complete cases for temporal validation. Detailed missing-data information for candidate predictors is presented in Supplementary Table S2, and the complete-case modeling datasets are summarized in Supplementary Table S5.

### 2.3 Prediction target, index date, and follow-up

The primary outcome was delivery of at least one live-born infant within 365 days after the index consultation. The index date was defined as the date of the first infertility consultation at which baseline clinical characteristics and basal endocrine measurements were available.

The 365-day horizon was selected *a priori* to provide a fixed and clinically interpretable near-term prognostic window from the initial infertility consultation and to ensure a uniform period of outcome ascertainment across cohorts. This endpoint captured delivery, rather than conception or pregnancy, within the prespecified 365-day period. Therefore, a pregnancy initiated within 365 days but resulting in delivery after day 365 was not counted as an outcome event.

Follow-up started on the index date and ended at delivery of a live-born infant, completion of the 365-day outcome window, loss to follow-up, or the administrative end of outcome ascertainment on December 31, 2025, whichever occurred first. Women without a recorded delivery of a live-born infant were classified as not having achieved the outcome only when pregnancy and delivery outcomes had been verified through day 365 after the index consultation. Women with missing outcome information or incomplete follow-up before day 365 were excluded from the primary analysis.

For analytic purposes, follow-up duration was calculated from the index date to delivery of a live-born infant or day 365, whichever occurred first. Thus, all women classified as not having achieved the outcome had complete outcome ascertainment through the prespecified 365-day prediction horizon.

#### 2.3.1 Clinical care pathway and intended use of the prediction model

After the index consultation, women received routine individualized infertility care at our center. Management decisions were made by reproductive specialists according to age, body mass index, ovulatory status, duration of infertility, reproductive history, tubal/pelvic and uterine factors, male-factor assessment, previous treatment response, and patient preferences. Depending on clinical indications, routine care could include lifestyle and weight-management counseling, ovulation induction, insulin-sensitizing treatment, gonadotropin therapy, intrauterine insemination, assisted reproductive technology, and treatment of coexisting uterine, tubal/pelvic, or male factors.

The present model was designed for prediction at the initial infertility assessment. Therefore, post-index treatment variables were not included as candidate predictors because they occurred after the prediction time point, were influenced by baseline patient characteristics and clinical decision-making, and were not uniformly available at sufficient detail for treatment-specific analysis. Accordingly, the model estimates the probability of delivery of a live-born infant within 365 days after the index consultation under the heterogeneous routine-care pathway at our center. It should not be interpreted as a treatment-specific prediction model or as evidence of the comparative effectiveness of different infertility treatments. For decision curve analysis, the event was defined as no delivery of a live-born infant within 365 days after the index consultation. Because the final logistic regression model estimated the probability of delivery of a live-born infant within 365 days, the predicted risk of non-live-birth was calculated as 1 minus the predicted probability of live birth.

A threshold probability represented the predicted risk of non-live-birth at which intensified prognostic counseling and further infertility evaluation would be considered appropriate. In this context, the treat-all strategy represented intensified counseling and further infertility evaluation for all women, whereas the treat-none strategy represented no risk-guided intensification of counseling or evaluation. These strategies were not intended to determine whether infertility treatment should be initiated, withheld, or replaced by a specific treatment pathway.

### 2.4 Cohort division

Patients with an index date between December 1, 2021, and December 31, 2023, were assigned to the development cohort. Patients with an index date between January 1, 2024, and December 31, 2024, were assigned to the same-center temporal validation cohort. The administrative outcome ascertainment date was December 31, 2025, ensuring that all eligible women enrolled in 2024, including those enrolled on December 31, 2024, had the opportunity to complete the 12-month follow-up window.

### 2.5 Model development and validation

A multivariable logistic regression model was used to develop a prediction model for live birth in infertile women with PCOS. Candidate predictors were prespecified before model development according to previous literature, routine clinical practice, and expert opinion. The initial candidate variables included female age, body mass index (BMI), infertility duration, type of infertility, gravidity, parity, miscarriage history, ectopic pregnancy history, basal endocrine indicators (FSH, LH, LH/FSH ratio, estradiol, progesterone, prolactin, and testosterone), tubal/pelvic factors, ovulatory dysfunction, uterine factors, male factors, thyroid disease, and abnormal glucose metabolism.

Univariable logistic regression analyses were performed to describe crude associations between each candidate predictor and live birth; however, statistical significance in univariable analysis was not used as the sole criterion for variable selection. Final predictors were selected primarily according to their prespecified clinical relevance, routine availability at the index consultation, acceptable data completeness, and contribution to overall model stability and clinical applicability.

No automated stepwise selection, penalized regression, or shrinkage methods were used. Variables with excessive missingness (e.g., AMH) were excluded before model development because reliable imputation was not considered appropriate in this retrospective dataset.

Model discrimination was evaluated using the receiver operating characteristic (ROC) curve and the area under the curve (*AUC*). Calibration was assessed using calibration curves, the Brier score, calibration intercept, and calibration slope. Clinical utility was evaluated using decision curve analysis. Internal validation was performed in the development cohort using bootstrap resampling. The complete-case development dataset included 349 women, including 158 women who delivered a live-born infant within 365 days after the index consultation. The final prediction model contained seven predictor parameters, corresponding to approximately 23 events per predictor parameter. Bootstrap resampling was used to internally validate the performance of the prespecified final model. Predictor selection was not repeated during each bootstrap sample; therefore, the bootstrap procedure estimated optimism in model performance for the fixed final model rather than evaluating variable-selection stability. The model developed in the development cohort was directly applied to the temporal validation cohort without re-estimating the model coefficients.

To facilitate independent implementation of the prediction model, the complete logistic regression equation, including the model intercept, regression coefficients, predictor coding, and probability calculation formula, is provided in Supplementary Table S1. Continuous predictors were entered into the model in their original units without transformation. Binary predictors were coded as 0 = absent and 1 = present, whereas gravidity was entered into the model as a continuous predictor per previous pregnancy. Individual predicted probabilities were calculated using the standard logistic regression equation:

$$P=\frac{1}{1+\exp\left[-\left(\beta_{0}+\sum \beta_{i}X_{i}\right)\right]}$$

where *P* represents the predicted probability of live birth within 365 days.

To assess the appropriateness of modeling continuous predictors as linear terms, restricted cubic spline analyses were additionally performed for female age, BMI, infertility duration, and LH/FSH ratio. Because no statistically significant departures from linearity were observed, these predictors were retained in their original linear form in the final prediction model. The nonlinearity test results are presented in Supplementary Table S3.

### 2.6 Statistical analysis

Continuous variables are presented as medians and interquartile ranges, and categorical variables are presented as frequencies and percentages. Baseline characteristics were compared between the development and temporal validation cohorts, as well as between patients with different live birth outcomes in the development cohort, using the Wilcoxon rank-sum test, Pearson's *χ*^2^ test, or Fisher's exact test, as appropriate. Univariable and multivariable logistic regression analyses were used to identify factors associated with live birth, and odds ratios (ORs), 95% confidence intervals (CIs), and *P* values were reported. Because the objective of this study was prediction rather than causal inference, clinically important variables were retained in the final model even if they were not statistically significant after multivariable adjustment.

Model discrimination was assessed using the area under the receiver operating characteristic curve (*AUC*). Calibration was evaluated using calibration curves, the Brier score, calibration intercept, and calibration slope. Clinical utility was assessed using decision curve analysis (DCA). For DCA, the event was coded as non-live-birth within 365 days after the index consultation, and the predicted risk was calculated as 1 minus the model-predicted probability of delivery of a live-born infant within 365 days. Internal validation was performed in the development cohort using bootstrap resampling, and the final model was directly applied to the temporal validation cohort without re-estimating the model coefficients. All statistical tests were two-sided, and *P* < 0.05 was considered statistically significant. Statistical analyses were performed using R software version 4.3.2, mainly with the rms, pROC, rmda, and gtsummary packages.

## 3 Results

### 3.1 Study population and cohort division

A total of 746 records were initially screened. After exclusion of 18 records that did not meet the PCOS/infertility inclusion criteria, 26 records without adequate baseline clinical assessment or basal endocrine testing, 72 records with missing or incomplete 365-day outcome ascertainment, 11 duplicate records, and 9 records with unverifiable data-entry errors, 610 women remained in the eligible analytic cohort.

The eligible analytic cohort comprised 389 women in the development cohort and 221 women in the same-center temporal validation cohort. Among the 610 women, 272 delivered a live-born infant within 365 days after the index consultation, including 174 women in the development cohort and 98 women in the temporal validation cohort.

For final model estimation, complete-case analysis was performed using the seven final predictors. In the development cohort, 40 women had missing data for at least one final predictor, leaving 349 complete cases, of whom 158 delivered a live-born infant within 365 days after the index consultation. In the temporal validation cohort, 18 women had missing data for at least one final predictor, leaving 203 complete cases, of whom 91 delivered a live-born infant within 365 days after the index consultation. All multivariable regression, discrimination, calibration, bootstrap validation, and decision curve analyses were based on these complete-case datasets. The administrative outcome ascertainment date was December 31, 2025. Overall, 272 women delivered a live-born infant within 365 days after the index consultation, whereas 338 women did not deliver a live-born infant within this prespecified period. All women classified as not having achieved live birth had complete 365-day outcome ascertainment. The median observed follow-up duration was 365 days (IQR, 315–365 days), and the minimum observed follow-up duration was 245 days. The minimum confirmed follow-up duration among women without live birth was 365 days. The proportions of women who delivered a live-born infant within 365 days after the index consultation were 44.7% in the development cohort and 44.3% in the same-center temporal validation cohort. Overall, the main clinical characteristics were relatively balanced between the two cohorts. Statistically significant differences were observed in estradiol (E2) levels, number of ectopic pregnancies, and distribution of uterine factors between the two cohorts. The difference in E2 levels may partly reflect variation in menstrual-cycle timing, amenorrhea-related sampling procedures, patient characteristics, or routine clinical workflow, despite the use of the same laboratory platform and standardized CLIA-based measurements. No statistically significant differences were found in other variables, including female age, body mass index (BMI), type of infertility, duration of infertility, luteinizing hormone/follicle-stimulating hormone (LH/FSH) ratio, tubal/pelvic factors, ovulatory dysfunction, thyroid disease, and abnormal glucose metabolism. The baseline characteristics of the development and temporal validation cohorts are presented in Table 1. Detailed missing-data information for candidate predictors and outcomes is presented in Supplementary Table S2.

**Table 1:** Baseline characteristics of the development and temporal validation cohorts.

Variable	Overall (*n* = 610)	Development cohort (*n* = 389)	Temporal validation cohort (*n* = 221)	*P* value
Female age, years	30.0 (27.0, 33.0)	31.0 (27.0, 33.0)	30.0 (27.0, 33.0)	0.2
BMI, kg/m^2^	25.7 (22.8, 28.3)	26.0 (22.8, 28.5)	25.2 (22.8, 28.0)	0.3
Type of infertility				0.7
Primary infertility	298 (49%)	188 (48%)	110 (50%)	
Secondary infertility	312 (51%)	201 (52%)	111 (50%)	
Duration of infertility, years	4.0 (2.0, 6.0)	4.0 (2.0, 6.0)	3.0 (2.0, 6.0)	0.14
Gravidity				0.3
0	269 (44%)	165 (42%)	104 (47%)	
1	205 (34%)	131 (34%)	74 (33%)	
2	70 (11%)	42 (11%)	28 (13%)	
≥3	66 (10.8%)	51 (13.1%)	15 (6.8%)	
Parity				0.9
0	442 (72%)	279 (72%)	163 (74%)	
≥1	168 (27.5%)	110 (28.3%)	58 (26.2%)	
Number of miscarriages				0.13
0	426 (70%)	267 (69%)	159 (72%)	
≥1	184 (30.2%)	122 (31.4%)	62 (28.1%)	
Number of ectopic pregnancies				0.024
0	570 (93.4%)	362 (93.1%)	208 (94.1%)	
1	28 (4.6%)	15 (3.9%)	13 (5.9%)	
2	10 (1.6%)	10 (2.6%)	0 (0.0%)	
3	2 (0.3%)	2 (0.5%)	0 (0.0%)	
FSH, mIU/mL	5.76 (4.81, 6.75)	5.77 (4.74, 6.75)	5.76 (4.89, 6.77)	0.4
LH, mIU/mL	7.8 (4.9, 11.9)	7.6 (4.8, 11.5)	8.6 (5.2, 12.2)	0.3
LH/FSH ratio	1.38 (0.90, 1.97)	1.35 (0.87, 1.94)	1.46 (0.95, 2.10)	0.2
E2, pg/mL	50 (38, 68)	46 (36, 62)	55 (44, 78)	<0.001
Progesterone, ng/mL	0.29 (0.17, 0.47)	0.29 (0.16, 0.45)	0.32 (0.19, 0.47)	0.082
PRL, ng/mL	260 (189, 342)	271 (179, 356)	255 (202, 310)	0.5
Testosterone, ng/mL	0.46 (0.33, 0.61)	0.44 (0.31, 0.60)	0.48 (0.35, 0.61)	0.2
Tubal/pelvic factors				0.6
No	289 (47%)	181 (47%)	108 (49%)	
Yes	321 (53%)	208 (53%)	113 (51%)	
Recorded ovulatory dysfunction				0.5
No	191 (31%)	118 (30%)	73 (33%)	
Yes	419 (69%)	271 (70%)	148 (67%)	
Uterine factors				0.037
No	511 (84%)	335 (86%)	176 (80%)	
Yes	99 (16%)	54 (14%)	45 (20%)	
Recorded male-factor infertility				0.5
No	602 (99%)	385 (99%)	217 (98%)	
Yes	8 (1.3%)	4 (1.0%)	4 (1.8%)	
Thyroid disease				0.2
No	578 (95%)	372 (96%)	206 (93%)	
Yes	32 (5.2%)	17 (4.4%)	15 (6.8%)	
Abnormal glucose metabolism				>0.900
No	583 (96%)	372 (96%)	211 (95%)	
Yes	27 (4.4%)	17 (4.4%)	10 (4.5%)	
Delivery of a live-born infant within 365 days after the index consultation				>0.900
No	338 (55%)	215 (55%)	123 (56%)	
Yes	272 (45%)	174 (45%)	98 (44%)	

Continuous variables are presented as median (Q1, Q3), and categorical variables are presented as *n* (%). *P* values were derived from the Wilcoxon rank-sum test, Pearson's *χ^2^* test, or Fisher's exact test. Recorded ovulatory dysfunction and recorded male-factor infertility were based on diagnoses documented during routine infertility evaluation; operational definitions are provided in the Methods.

### 3.2 Baseline characteristics according to live birth outcome in the development cohort

In the development cohort, compared with patients who did not achieve live birth, those who achieved live birth were younger [29.0 (26.0, 32.0) years vs. 32.0 (28.0, 35.0) years, *P* < 0.001], had a lower BMI [25.2 (22.1, 28.0) kg/m^2^ vs. 26.3 (23.7, 28.9) kg/m^2^, *P* = 0.007], and had a shorter duration of infertility [3.00 (2.00, 4.00) years vs. 5.00 (3.00, 7.00) years, *P* < 0.001]. In addition, statistically significant differences were observed between the two groups in gravidity, parity, number of miscarriages, and distribution of uterine factors. The proportion of uterine factors was markedly lower in the live birth group than in the non-live birth group (6.9% vs. 20%, *P* < 0.001). Detailed baseline characteristics stratified by live birth outcome are presented in Table 2.

**Table 2:** Baseline characteristics of the development cohort stratified by live birth outcome.

Variable	Overall (*n* = 389)	No live birth (*n* = 215)	Live birth (*n* = 174)	*P* value
Female age, years	31.0 (27.0, 33.0)	32.0 (28.0, 35.0)	29.0 (26.0, 32.0)	<0.001
BMI, kg/m^2^	26.0 (22.8, 28.5)	26.3 (23.7, 28.9)	25.2 (22.1, 28.0)	0.007
Type of infertility				0.200
Primary infertility	188 (48%)	97 (45%)	91 (52%)	
Secondary infertility	201 (52%)	118 (55%)	83 (48%)	
Duration of infertility, years	4.00 (2.00, 6.00)	5.00 (3.00, 7.00)	3.00 (2.00, 4.00)	<0.001
Gravidity				0.040
0	165 (42%)	76 (35%)	89 (51%)	
1	131 (34%)	81 (38%)	50 (29%)	
2	42 (11%)	28 (13%)	14 (8.0%)	
≥3	51 (13.1%)	30 (14.0%)	21 (12.1%)	
Parity				0.015
0	279 (72%)	142 (66%)	137 (79%)	
≥1	110 (28.3%)	73 (34.0%)	37 (21.3%)	
Number of miscarriages				0.015
0	267 (69%)	138 (64%)	129 (74%)	
≥1	122 (31.4%)	77 (35.8%)	45 (25.9%)	
Number of ectopic pregnancies				0.068
0	362 (93%)	206 (96%)	156 (90%)	
≥1	27 (6.9%)	9 (4.2%)	18 (10.3%)	
FSH	5.77 (4.74, 6.75)	5.60 (4.51, 6.64)	5.98 (4.98, 6.86)	0.094
LH	7.6 (4.8, 11.5)	7.5 (4.8, 11.9)	7.6 (4.9, 11.3)	>0.900
LH/FSH ratio	1.35 (0.87, 1.94)	1.37 (0.86, 1.98)	1.27 (0.87, 1.85)	0.300
E2	46 (36, 62)	47 (37, 65)	45 (36, 57)	0.200
P	0.29 (0.16, 0.45)	0.29 (0.15, 0.48)	0.29 (0.18, 0.43)	0.700
PRL, ng/mL	271 (179, 356)	250 (179, 356)	286 (179, 351)	0.200
T, ng/mL	0.44 (0.31, 0.60)	0.45 (0.31, 0.59)	0.42 (0.33, 0.61)	0.900
Tubal/pelvic factors				0.800
No	181 (47%)	99 (46%)	82 (47%)	
Yes	208 (53%)	116 (54%)	92 (53%)	
Recorded ovulatory dysfunction				0.068
No	118 (30%)	57 (27%)	61 (35%)	
Yes	271 (70%)	158 (73%)	113 (65%)	
Uterine factors				<0.001
No	335 (86%)	173 (80%)	162 (93%)	
Yes	54 (14%)	42 (20%)	12 (6.9%)	
Recorded male-factor infertility				>0.900
No	385 (99%)	213 (99%)	172 (99%)	
Yes	4 (1.0%)	2 (0.9%)	2 (1.1%)	
Thyroid disease				0.800
No	372 (96%)	205 (95%)	167 (96%)	
Yes	17 (4.4%)	10 (4.7%)	7 (4.0%)	
Abnormal glucose metabolism				0.400
No	372 (96%)	204 (95%)	168 (97%)	
Yes	17 (4.4%)	11 (5.1%)	6 (3.4%)	

Continuous variables are presented as median (Q1, Q3), and categorical variables are presented as *n* (%). *P* values were derived from the Wilcoxon rank-sum test, Pearson's *χ*^2^ test, or Fisher's exact test. Recorded ovulatory dysfunction and recorded male-factor infertility were based on diagnoses documented during routine infertility evaluation; operational definitions are provided in the Methods.

### 3.3 Univariable logistic regression analysis

Univariable logistic regression analysis in the development cohort showed that older age (OR = 0.88, 95% CI: 0.83–0.93, *P* < 0.001), higher BMI (OR = 0.92, 95% CI: 0.87–0.97, *P* = 0.002), longer duration of infertility (OR = 0.80, 95% CI: 0.73–0.87, *P* < 0.001), and higher gravidity (OR = 0.82, 95% CI: 0.67–0.98, *P* = 0.036) were associated with a lower probability of live birth. The probability of live birth was also significantly reduced in patients with uterine factors (OR = 0.24, 95% CI: 0.11–0.50, *P* < 0.001). The complete univariable logistic regression results are presented in Table 3.

**Table 3:** Univariable logistic regression analysis of factors associated with live birth outcome in the development cohort.

Variable	OR	95% CI	*P* value
Female age, years	0.88	0.83–0.93	<0.001
BMI, kg/m^2^	0.92	0.87–0.97	0.002
Duration of infertility, years	0.8	0.73–0.87	<0.001
Gravidity	0.82	0.67–0.98	0.036
LH/FSH ratio	0.87	0.69–1.07	0.2
Tubal/pelvic factors: yes vs. no	1	0.66–1.53	>0.900
Uterine factors: yes vs. no	0.24	0.11–0.50	<0.001

OR, odds ratio; CI, confidence interval.

### 3.4 Multivariable logistic regression prediction model

Multivariable logistic regression analysis showed that age, BMI, duration of infertility, LH/FSH ratio, and uterine factors were important predictors of live birth outcome. For each 1-year increase in age, the odds of live birth decreased by approximately 9% (OR = 0.91, 95% CI: 0.85–0.98, *P* = 0.008). For each 1 kg/m^2^ increase in BMI, the odds of live birth decreased by approximately 8% (OR = 0.92, 95% CI: 0.86–0.98, *P* = 0.008). For each 1-year increase in the duration of infertility, the odds of live birth decreased by approximately 15% (OR = 0.85, 95% CI: 0.77–0.94, *P* = 0.002). A higher LH/FSH ratio was also associated with a lower probability of live birth (OR = 0.71, 95% CI: 0.55–0.91, *P* = 0.009). Patients with uterine factors had a significantly lower probability of live birth (OR = 0.37, 95% CI: 0.15–0.80, *P* = 0.016). Although gravidity and tubal/pelvic factors did not reach statistical significance in the multivariable model, they were retained in the prediction model because of their clear clinical relevance. The complete multivariable logistic regression model is presented in Table 4. The complete regression equation, predictor coding, regression coefficients, intercept, and an example of probability calculation are presented in Supplementary Table S1 to facilitate independent application of the model.

**Table 4:** Multivariable logistic regression model for delivery of a live-born infant within 365 days after the index consultation in the complete-case development cohort (*n* = 349; 158 events).

Variable	OR	95% CI	*P* value
Female age, years	0.91	0.85–0.98	0.008
BMI, kg/m^2^	0.92	0.86–0.98	0.008
Duration of infertility, years	0.85	0.77–0.94	0.002
Gravidity	0.91	0.73–1.12	0.400
LH/FSH ratio	0.71	0.55–0.91	0.009
Tubal/pelvic factors: yes vs. no	0.78	0.48–1.26	0.300
Uterine factors: yes vs. no	0.37	0.15–0.80	0.016

The multivariable analysis was conducted in 349 complete cases from the development cohort, including 158 live-birth events.

### 3.5 Assessment of linearity

Restricted cubic spline analyses showed no statistically significant departures from linearity for age (*P for nonlinearity* = 0.059), BMI (*P* = 0.360), infertility duration (*P* = 0.983), or LH/FSH ratio (*P* = 0.944). Therefore, all continuous predictors were retained as linear terms in the final prediction model. The nonlinearity test results are presented in Supplementary Table S3.

### 3.6 Summary of model performance

The final prediction model achieved an *AUC* of 0.735 (95% CI: 0.682–0.787) in the development cohort and 0.738 (95% CI: 0.670–0.807) in the temporal validation cohort, indicating moderate discrimination in the later same-center temporal validation cohort. However, the relatively wide confidence interval suggests that the precision of the validation performance remains limited. The Brier scores were 0.208 and 0.205 in the development and temporal validation cohorts, respectively. In the temporal validation cohort, the calibration intercept was −0.077 and the calibration slope was 0.896, suggesting acceptable overall calibration performance.

#### 3.6.1 Sensitivity analysis

A sensitivity analysis was performed by refitting a reduced model after excluding gravidity and tubal/pelvic factors. Compared with the final model, the reduced model showed slightly lower discrimination in both the development cohort (AUC: 0.725 vs. 0.735) and the temporal validation cohort (AUC: 0.731 vs. 0.738). Brier scores differed only minimally between the two models (Supplementary Table S4). Considering the modest reduction in discrimination together with the established clinical relevance of these variables, the original model was retained.

### 3.7 Development of the prediction model and presentation of the nomogram

**Figure 1:**
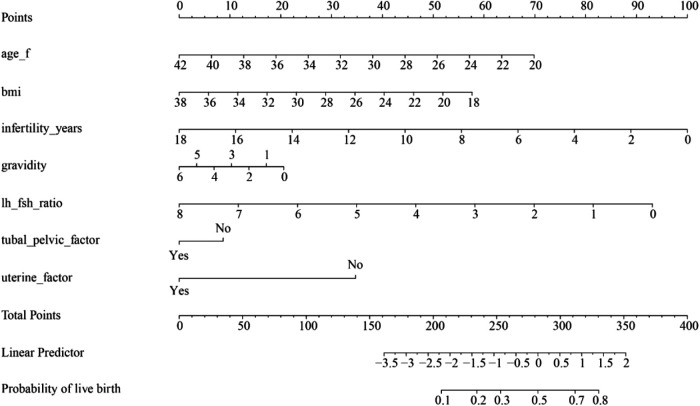
Nomogram for predicting the probability of delivery of a live-born infant within 365 days after the index consultation in infertile women with a documented diagnosis of polycystic ovary syndrome.

A prediction model for live birth outcome in infertile women with PCOS was developed based on the development cohort, and a nomogram was further constructed. The final model included female age, BMI, duration of infertility, gravidity, LH/FSH ratio, tubal/pelvic factors, and uterine factors. The nomogram indicated that younger age, lower BMI, shorter duration of infertility, lower LH/FSH ratio, and absence of uterine factors were associated with higher total scores and a relatively higher predicted probability of live birth. The total score can be calculated by summing the points assigned to each predictor, which can then be used to estimate the individualized probability of live birth (Figure 1). The nomogram was directly derived from the underlying multivariable logistic regression model, the full mathematical equation of which is provided in Supplementary Table S1.

The nomogram was derived from the complete-case development cohort (*n* = 349; 158 women delivered a live-born infant within 365 days after the index consultation).

### 3.8 Evaluation of model discrimination

**Figure 2:**
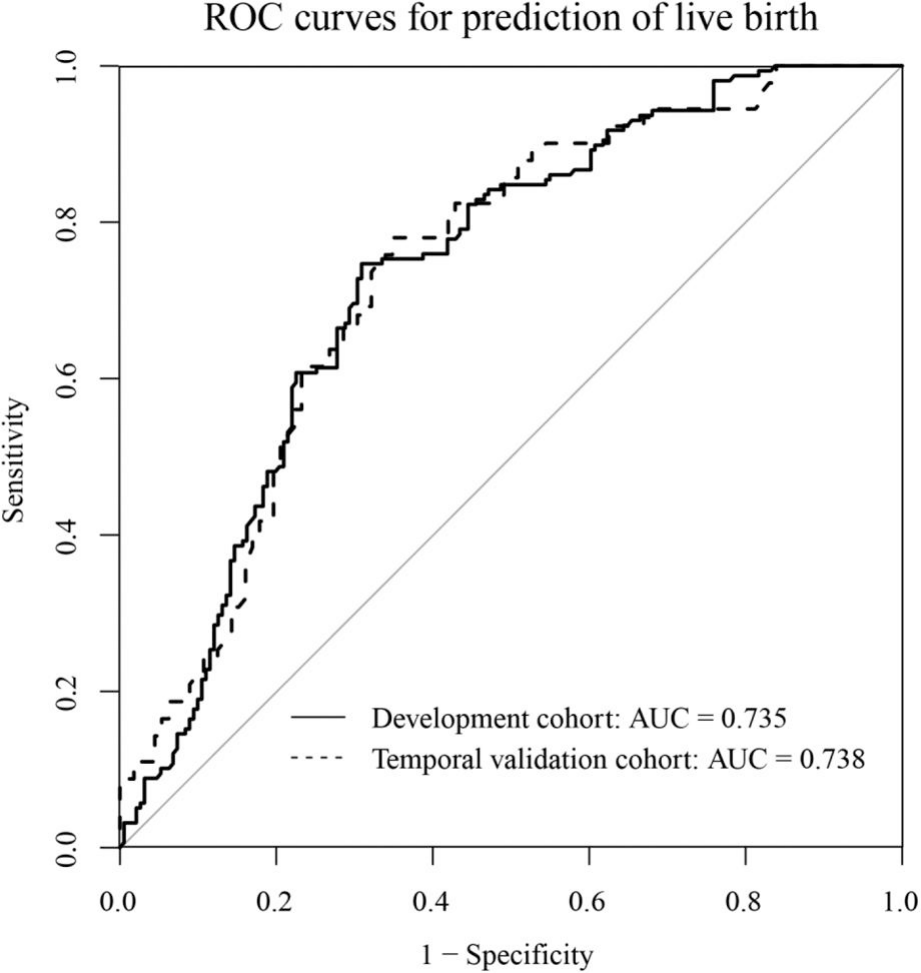
Receiver operating characteristic curves for the prediction model in the development and temporal validation cohorts.

Receiver operating characteristic curve analysis showed that the model had moderate discrimination in the development cohort, with an *AUC* of 0.735 (95% CI: 0.682–0.787). When the model derived from the development cohort was directly applied to the temporal validation cohort, the *AUC* was 0.738 (95% CI: 0.670–0.807), which was comparable to that in the development cohort. These findings suggest that the model maintained relatively stable predictive performance in patients from a later period. The ROC curves are shown in Figure 2.

The ROC analysis was conducted in the complete-case development cohort (*n* = 349; 158 live-birth events) and the complete-case temporal validation cohort (*n* = 203; 91 live-birth events).

### 3.9 Evaluation of model calibration

**Figure 3:**
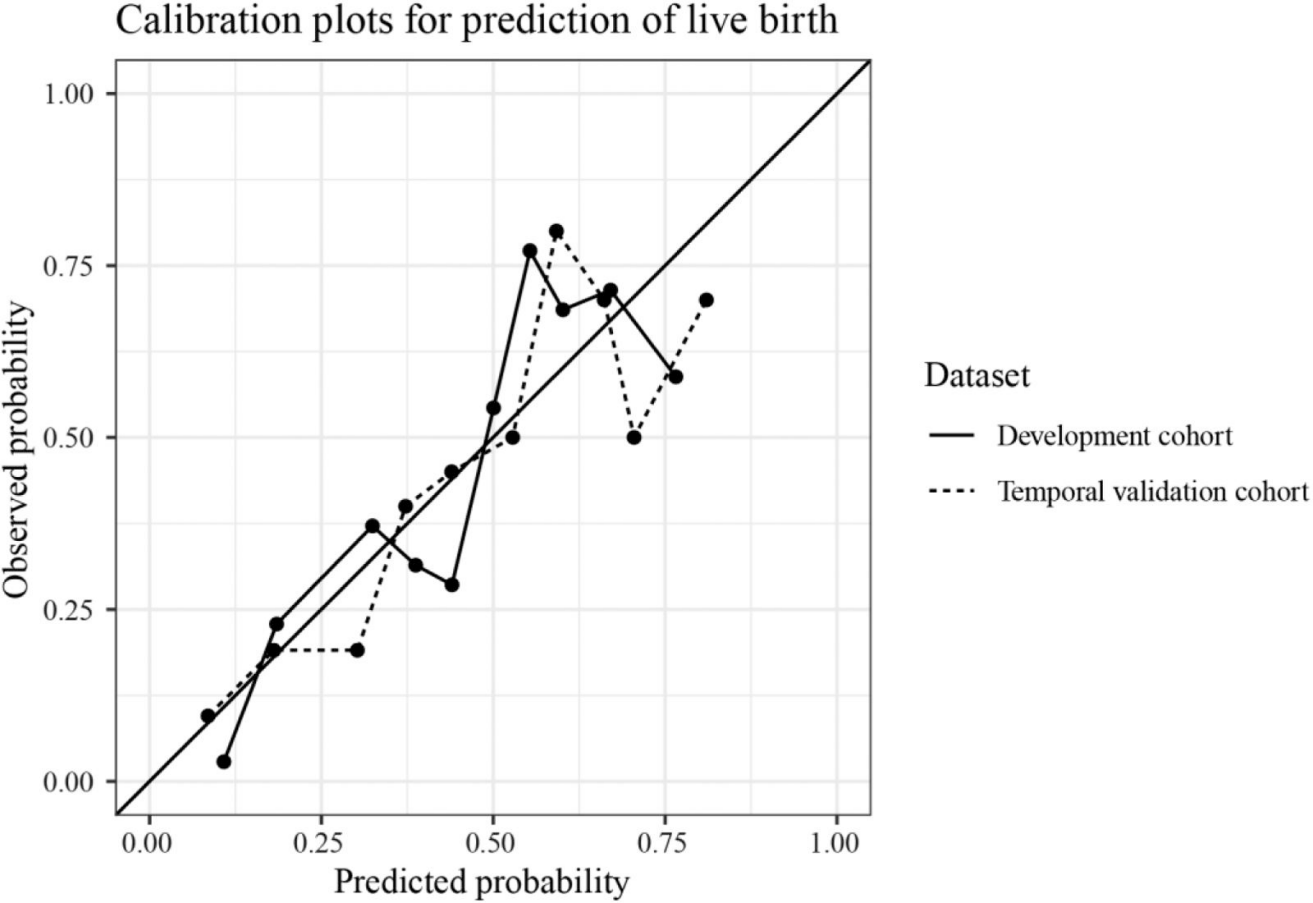
Calibration plots of the prediction model for live birth.

The calibration curve showed that the predicted probabilities generated by the model were generally close to the observed probabilities of live birth in the development cohort. The bootstrap-corrected calibration curve showed an overall trend consistent with the ideal reference line, with a mean absolute error of 0.032, indicating good internal calibration performance in the development cohort. Model calibration was further evaluated in the temporal validation cohort. The calibration intercept was −0.077, the calibration slope was 0.896, and the Brier score was 0.205, suggesting acceptable overall calibration, although the calibration slope below 1 indicates mild overfitting and slightly overconfident predictions. The calibration curves are shown in Figure 3.

The calibration curves showed acceptable agreement between predicted and observed probabilities in both the development and temporal validation cohorts. The bootstrap-corrected calibration curve in the development cohort showed a mean absolute error of 0.032. Calibration was assessed in the complete-case development cohort (*n* = 349; 158 live-birth events) and the complete-case temporal validation cohort (*n* = 203; 91 live-birth events).

### 3.10 Decision curve analysis

Decision curve analysis was performed using non-live-birth within 365 days after the index consultation as the event. The predicted risk of non-live-birth was calculated as 1 minus the model-predicted probability of delivery of a live-born infant within 365 days.

The prediction model showed greater net benefit than the treat-all and treat-none strategies across threshold probabilities of approximately 0.37–0.82 in both the development and temporal validation cohorts. An additional net-benefit range was observed at higher threshold probabilities in both cohorts; however, the primary interpretation focused on the continuous range of 0.37–0.82.

**Figure 4:**
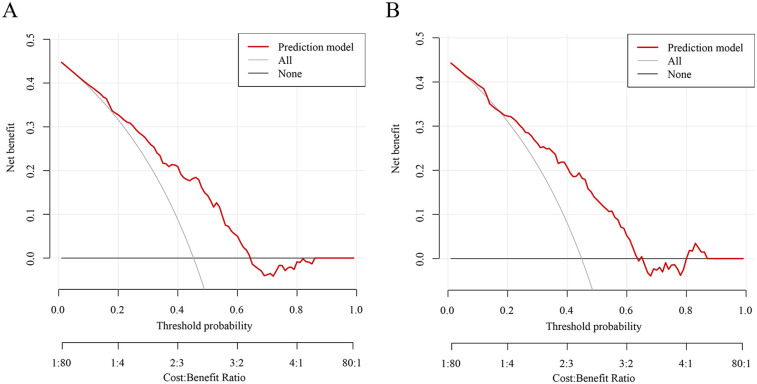
Decision curve analysis for the predicted risk of non-live-birth within 365 days after the index consultation. **(A)** Development cohort. **(B)** Temporal validation cohort. The event was defined as no delivery of a live-born infant within 365 days after the index consultation. The predicted risk of non-live-birth was calculated as 1 minus the model-predicted probability of delivery of a live-born infant within 365 days. A threshold probability indicates the predicted risk of non-live-birth at which intensified prognostic counseling and further infertility evaluation would be considered. Treat-all indicates intensified counseling and further evaluation for all women, whereas treat-none indicates no risk-guided intensification of counseling or evaluation. These strategies do not represent initiation, withholding, or selection of a specific infertility treatment.

In this analysis, a threshold probability represented the predicted risk of non-live-birth at which intensified prognostic counseling and further infertility evaluation would be considered appropriate. The treat-all strategy represented intensified counseling and further evaluation for all women, whereas the treat-none strategy represented no risk-guided intensification of counseling or evaluation. The analysis was not intended to guide initiation, withholding, or selection of a specific infertility treatment. The decision curve analysis results for the development and temporal validation cohorts are shown in Figure 4.

## 4 Discussion

Based on data from a single-center retrospective cohort, this study developed and temporally validated a clinical prediction model for live birth outcomes in infertile women with a documented diagnosis of polycystic ovary syndrome (PCOS). Live birth was selected as the primary outcome, which better reflects the final reproductive benefit for patients than biochemical pregnancy or clinical pregnancy alone. The final model included routinely available clinical and basal endocrine indicators, including female age, BMI, duration of infertility, gravidity, LH/FSH ratio, tubal/pelvic factors, and uterine factors. The final prediction model incorporated age, BMI, infertility duration, gravidity, LH/FSH ratio, tubal/pelvic factors, and uterine factors to estimate the probability of delivery of a live-born infant within 365 days after the index consultation. These variables should be interpreted as predictors contributing to individualized prognostic estimation rather than as causal determinants of reproductive outcomes because the present study was retrospective and designed for prediction rather than causal inference. Accordingly, the observed associations should not be interpreted as evidence that modifying an individual predictor would necessarily change the probability of delivery of a live-born infant within 365 days after the index consultation. The *AUC*s of the model were 0.735 and 0.738 in the development and temporal validation cohorts, respectively. The model showed moderate discrimination in both the development and same-center temporal validation cohorts. Because the validation cohort was derived from the same institution, these findings provide preliminary evidence of temporal reproducibility rather than external generalizability.

Older age was associated with a lower predicted probability of delivery of a live-born infant within 365 days after the index consultation in this cohort. Age may therefore serve as a routinely available prognostic marker during the initial infertility assessment. Although biological mechanisms related to reproductive aging have been described in previous studies (16–18), the present retrospective prediction model was not designed to determine whether age itself causally changes reproductive outcomes or whether altering the timing of care would change an individual patient's predicted outcome. Thus, age should be interpreted as contributing prognostic information for counseling and risk stratification rather than as a causal treatment target.

Higher BMI was associated with a lower predicted probability of delivery of a live-born infant within 365 days after the index consultation in this cohort. BMI may therefore provide useful prognostic information at the initial infertility assessment. However, this observational prediction model was not designed to estimate the causal effect of BMI or weight reduction on reproductive outcomes. Although weight management may be clinically appropriate as part of routine care for some women and has been evaluated in separate interventional studies (19, 20), the present findings do not demonstrate that reducing BMI would necessarily improve an individual patient's predicted probability of delivery of a live-born infant within 365 days. The present findings support the inclusion of BMI as a baseline prognostic marker in reproductive outcome risk stratification.

Longer duration of infertility was another stable unfavorable predictor in this study. Multivariable analysis showed that each 1-year increase in infertility duration was associated with an approximately 15% reduction in the odds of live birth. A longer duration of infertility may indicate more complex or persistent reproductive disorders, such as long-term ovulatory dysfunction, pelvic or tubal factors, abnormal endometrial environment, or poor response to previous treatment. It may also reflect delayed standardized diagnosis and treatment, or multiple previous treatment cycles without an ideal outcome. Therefore, in infertile women with PCOS, infertility duration is not merely an indicator of disease course, but may also comprehensively reflect disease complexity and treatment difficulty. Clinically, for patients with a longer duration of infertility, etiological reassessment should be strengthened, prolonged repetition of low-efficiency treatment should be avoided, and treatment strategies should be adjusted in a timely manner according to patient age, ovarian reserve, and coexisting infertility factors.

A higher LH/FSH ratio was associated with a lower predicted probability of delivery of a live-born infant within 365 days after the index consultation. The LH/FSH ratio may provide prognostic information as a composite marker of basal endocrine status at the initial infertility assessment. However, the observed association should not be interpreted as evidence that the LH/FSH ratio itself causally determines reproductive outcome or that modifying the ratio would necessarily change the predicted probability of delivery of a live-born infant. The finding supports the inclusion of routinely available basal endocrine information in prognostic assessment, rather than the use of the LH/FSH ratio as a treatment target.

The presence of uterine factors was associated with a lower predicted probability of delivery of a live-born infant within 365 days after the index consultation. This finding indicates that documented uterine factors may provide clinically relevant baseline prognostic information in this real-world infertility cohort. However, because the present study was observational and post-index treatment information was heterogeneous, the association should not be interpreted as evidence that a specific uterine factor causally reduces the probability of live birth or that treatment of a uterine factor would necessarily improve the predicted outcome. Uterine assessment may be considered as part of routine infertility evaluation according to clinical indications, but this prediction model does not determine the benefit of a specific intervention.

It is worth noting that gravidity and tubal/pelvic factors did not reach statistical significance in the multivariable model but were still retained in the final prediction model. This approach is reasonable. Prediction model development is not fully equivalent to causal inference; rather, its goal is to integrate multiple clinically available variables to improve individualized predictive performance. Regarding gravidity, the estimated odds ratio in the multivariable model was below 1, indicating that a greater number of previous pregnancies was not associated with a higher predicted probability of live birth in this cohort. This finding should not be interpreted as evidence that previous pregnancy reduces the chance of future live birth. Rather, among women with infertility and PCOS, higher gravidity may reflect a more complex reproductive history, including previous miscarriages, ectopic pregnancies, biochemical pregnancies, or unsuccessful pregnancies that did not result in live birth. Therefore, gravidity in this model should be regarded as a marker of reproductive history that may contribute to prediction, rather than as a causal determinant of reproductive prognosis. For tubal/pelvic factors, the OR in the multivariable model was 0.78 (95% CI: 0.48–1.26), suggesting a decreased odds of live birth among patients with tubal/pelvic factors, although the association did not reach statistical significance because of the relatively wide confidence interval and limited sample size. Nevertheless, tubal/pelvic factors were retained as clinically relevant baseline characteristics that may contribute to prognostic assessment. Their inclusion in the model should not be interpreted as a recommendation for any specific infertility treatment pathway. Therefore, this variable should not be excluded solely on the basis of its *P* value. Retaining these two variables may improve the clinical interpretability and applicability of the model. This prespecified selection strategy reduces the risk of instability associated with purely data-driven variable selection and improves the clinical interpretability of the prediction model. Additional sensitivity analysis demonstrated that excluding gravidity and tubal/pelvic factors resulted in a slight reduction in model discrimination without improving overall predictive performance. Therefore, these variables were retained because prediction models should prioritize overall predictive performance and clinical interpretability rather than statistical significance alone (Supplementary Table S4).

Regarding model performance, the *AUC*s of the model were approximately 0.74 in both the development and temporal validation cohorts, indicating moderate discrimination. For live birth outcomes in infertile women with PCOS, the final outcome is influenced by multiple factors, including ovarian function, sperm factors, embryo quality, endometrial receptivity, treatment regimen, adherence, and pregnancy complications. Therefore, it is difficult to achieve extremely high predictive accuracy using only routine clinical and basal endocrine indicators. In this context, the moderate discrimination achieved by the present model is clinically reasonable. The AUCs were similar in the development and same-center temporal validation cohorts, suggesting that the model maintained comparable discrimination in patients treated during a later period at the same institution. However, because the validation cohort originated from the same center, these findings should be interpreted as evidence of temporal reproducibility rather than external validity. Furthermore, the relatively wide confidence interval of the validation AUC indicates that the precision of the performance estimate remains limited. Therefore, the present findings should be considered preliminary until validated in independent multicenter populations with different clinical practices and patient characteristics.

The calibration results showed that the calibration intercept and calibration slope in the temporal validation cohort were −0.077 and 0.896, respectively, suggesting acceptable overall agreement between predicted and observed risks. However, the calibration slope below 1 may indicate mild overfitting or slightly overconfident predictions; therefore, recalibration or model updating may be required before application in external populations. The mean absolute error of the bootstrap-corrected calibration curve was 0.032, supporting good internal calibration performance. Decision curve analysis showed that the model provided favorable net benefit within a threshold probability range of approximately 0.37–0.82 in both the development and temporal validation cohorts, indicating that it may be useful for risk-guided prognostic counseling and further infertility evaluation across this clinically relevant range. Decision curve analysis suggested that the model may provide clinical value when used for preliminary prognostic counseling, early risk stratification, and planning of further infertility evaluation at the initial infertility assessment. A higher predicted risk of non-live-birth may prompt more comprehensive assessment of relevant clinical factors and expectation counseling. However, the model should not be interpreted as a recommendation to initiate, withhold, compare, or select a specific infertility treatment. Women with a relatively high predicted risk of non-live-birth may be considered for more comprehensive prognostic counseling and further infertility evaluation according to routine clinical indications. The model does not determine whether a specific factor should be modified or whether a particular infertility treatment should be selected. Conversely, women with a relatively high predicted probability may continue routine individualized management while receiving realistic counseling regarding their expected prognosis. Therefore, the threshold probabilities evaluated in the decision curve analysis should be interpreted as reflecting different clinical preferences for management intensity rather than absolute treatment thresholds.

Several prediction models for reproductive outcomes in women with PCOS have previously been reported. However, many were developed in selected populations undergoing ovulation induction or assisted reproductive technology and incorporated treatment-related variables, ovarian reserve markers, or laboratory parameters that are unavailable at the initial consultation. In contrast, the present model was specifically designed for use at the first infertility assessment using routinely available clinical characteristics and basal endocrine measurements obtained before treatment initiation. Furthermore, unlike many previous studies that relied solely on internal validation or random split-sample validation, the current study additionally performed temporal validation using a later patient cohort, providing preliminary evidence of model reproducibility over time. Nevertheless, external multicenter validation remains necessary before widespread clinical implementation. The novelty of the present study lies in developing a pretreatment prediction model based exclusively on routinely available clinical and basal endocrine variables collected at the initial infertility assessment and evaluating its performance using temporal validation. Rather than replacing clinical judgment, the model is intended to facilitate preliminary individualized prognostic counseling, early risk stratification, and planning of further infertility evaluation in routine clinical practice.

This study has several strengths. First, live birth was used as the primary outcome rather than biochemical pregnancy or clinical pregnancy alone, which better reflects the final treatment goal of both infertile patients and clinicians. Second, the variables included in the model were derived from routine clinical data and basal endocrine tests, making them easy to obtain, low in cost, and suitable for clinical implementation. Third, this study evaluated model performance using a temporal validation cohort, which better reflects the model's performance in subsequent patients than random split-sample validation or internal validation alone. Fourth, model performance was evaluated from multiple perspectives, including discrimination, calibration, and clinical net benefit, resulting in a more comprehensive assessment. Additional restricted cubic spline analyses supported the use of linear terms for the continuous predictors included in the final model, thereby maintaining model simplicity without an apparent loss of predictive performance.

This study has several limitations. First, the predicted probability represents the likelihood of delivery of at least one live-born infant within 365 days after the index consultation under the heterogeneous routine-care pathway at our center, rather than cumulative live birth over a longer treatment period. Because this endpoint required delivery within the prespecified 365-day window, the model may underestimate longer-term reproductive prognosis for women who conceive later in the treatment pathway or who conceive within 365 days but deliver after day 365. Therefore, the model should not be interpreted as predicting cumulative live birth beyond the prespecified 365-day horizon. Second, treatment selection after the index consultation may have been influenced by baseline patient characteristics, clinician judgment, treatment response, financial considerations, and patient preferences. Because detailed information on treatment category, treatment intensity, number of cycles, adherence, and cycle-level assisted reproductive treatment data was not uniformly available, treatment-specific models could not be developed. Accordingly, the model should be used for preliminary prognostic counseling, early risk stratification, and planning of further infertility evaluation, rather than for selecting a specific treatment pathway or comparing the effectiveness of different treatments. Third, this was a single-center retrospective study. Although same-center temporal validation was performed, it cannot replace true multicenter external validation. The transportability of the model across different hospitals, geographic regions, laboratory practices, and infertility treatment pathways remains uncertain. Fourth, several potentially important reproductive medicine variables were not included, such as ovulation-induction regimens, medication dosage, follicular development, endometrial thickness, ovulation-monitoring results, detailed semen parameters, embryo quality, and type of assisted reproductive treatment. Information on recent hormonal medication exposure and standardized washout before basal endocrine testing was not uniformly available in the retrospective records; therefore, residual medication-related variation in basal endocrine measurements cannot be excluded. Fifth, AMH was excluded from candidate predictor consideration because of substantial missingness in routine clinical records. In addition, complete-case analysis for the final model excluded women with missing values for one or more final predictors and may therefore have introduced selection bias. Sixth, diagnosis-related variables were extracted from medical-record diagnosis fields and may have been subject to incomplete documentation or classification errors. PCOS status was identified from routine clinical records, and standardized retrospective reassessment of all diagnostic features, exclusion criteria, and PCOS phenotypes was not feasible for every participant. Seventh, women with concomitant infertility-related conditions were intentionally retained to reflect routine clinical practice. Therefore, the model estimates prognosis in a real-world infertility population with a documented diagnosis of PCOS rather than in a highly selected population in whom PCOS is the sole documented infertility-related condition. Caution is warranted when applying the model to highly selected populations in whom PCOS is considered the sole infertility diagnosis. Finally, the model was developed for prediction rather than causal inference and should not be used to infer causal relationships. Although it demonstrated stable performance in a temporally separated cohort from the same institution, its discrimination remained moderate, with an AUC of approximately 0.74. Therefore, the nomogram should be regarded as a tool for preliminary individualized prognostic counseling and early risk stratification at the initial infertility assessment, rather than as a standalone decision-making instrument. Multicenter external validation and prospective impact studies are needed before broader clinical implementation.

Future studies should further validate and update this model in multicenter, larger-scale, and prospective cohorts. Subsequent models may integrate ovarian reserve indicators, ovulation-induction treatment information, ultrasound monitoring parameters, endometrial receptivity indicators, male factors, and assisted reproductive laboratory indicators to improve predictive performance. An online calculator or clinical scoring tool based on an externally validated model could be developed to support outpatient prognostic counseling, early risk stratification, and planning of further infertility evaluation. Separate treatment-specific prediction models would require dedicated datasets with standardized and detailed treatment information and should be developed and validated independently.

This prediction model estimates the probability of delivery of a live-born infant within 365 days after the initial infertility consultation using routinely available clinical variables. The model demonstrated moderate discrimination and acceptable calibration in a same-center temporal validation cohort. It may support preliminary individualized prognostic counseling, early risk stratification, and planning of further infertility evaluation, but should not be used to select, withhold, or compare specific infertility treatments. Multicenter external validation and prospective impact studies are required before broader clinical implementation.

## Data Availability

The datasets presented in this article are not readily available because the individual-level clinical data generated and/or analyzed during the current study are not publicly available because of privacy and ethical restrictions. Reasonable requests for access to de-identified data may be considered by the corresponding author, subject to approval by the institutional ethics committee and completion of applicable data-use requirements. Requests to access the datasets should be directed to Yingjie Zhou, PhD, Hebei Reproductive Health Hospital, yanyuewuhen1@163.com.

## Supplementary material

Supplementary Figure S1 Participant flow diagram for cohort formation and complete-case model analyses.

Supplementary Table S1 Complete logistic regression equation and predictor coding of the final prediction model.

Supplementary Table S2 Missing data for candidate predictors and outcomes.

Supplementary Table S3 Restricted cubic spline assessment of nonlinearity for continuous predictors.

Supplementary Table S4 Sensitivity analysis comparing the full prediction model with a reduced model excluding gravidity and tubal/pelvic factors.

Supplementary Table S5 Complete-case datasets used for final model estimation and temporal validation.

## References

[1] Teede HJ Khomami MB Morman R Laven JSE Joham AE Costello MF. Polyendocrine metabolic ovarian syndrome, the new name for polycystic ovary syndrome: a multistep global consensus process. Lancet. (2026) 407(10545):2329–39. 10.1016/S0140-6736(26)00717-8 42119588

[2] Endocrine Society. PCOS name change: polycystic ovary syndrome renamed polyendocrine metabolic ovarian syndrome (2026). Available online at: https://www.endocrine.org/news-and-advocacy/news-room/2026/pcos-name-change (Accessed July 16, 2026).

[3] Salari N Nankali A Ghanbari A Jafarpour S Ghasemi H Dokaneheifard S. Global prevalence of polycystic ovary syndrome in women worldwide: a comprehensive systematic review and meta-analysis. Arch Gynecol Obstet. (2024) 310(3):1303–14. 10.1007/s00404-024-07607-x 38922413

[4] Teede HJ Tay CT Laven JJE Dokras A Moran LJ Piltonen TT. Recommendations from the 2023 international evidence-based guideline for the assessment and management of polycystic ovary syndrome. Eur J Endocrinol. (2023) 189(2):G43–64. 10.1093/ejendo/lvad096 37580861

[5] Wen X Wang L Lv S. Follicular development and endometrial receptivity of different androgen phenotypes in women with polycystic ovary syndrome. Front Endocrinol. (2024) 15:1400880. 10.3389/fendo.2024.1400880 PMC11669509 39726841

[6] Szkodziak P Szkodziak F Trzeciak K Woźniak S Mlynarczyk M Paszkowski T. Insulin resistance in polycystic ovary syndrome phenotypes and the vicious cycle model in its etiology. Sci Rep. (2025) 15(1):42649. 10.1038/s41598-025-26718-2 41315368 PMC12663220

[7] Guo Y Yang J Chen H Zhou Y Yang Y Wang B. Enhancing understanding of endometrial function in patients with PCOS: clinical and immunological insights. J Ovarian Res. (2025) 18(1):52. 10.1186/s13048-025-01638-x 40075432 PMC11900192

[8] Liang Y Zhang Q Lou Z. Effect of pre-treatment with oral short-acting contraceptives on assisted reproductive technology outcomes in patients with polycystic ovary syndrome: a meta-analysis. Front Endocrinol. (2025) 16:1545508. 10.3389/fendo.2025.1545508 PMC12176574 40538799

[9] Cooney LG Sammel MD Lee I Clapp MA Goldsammler M Scott E. The details matter: personalized prediction of live birth after in vitro fertilization in women with polycystic ovary syndrome. Fertil Steril. (2024) 121(6):1010–9. 10.1016/j.fertnstert.2024.01.033 38307452

[10] Leal CRV Zanolla K Spritzer PM Reis FM. Assisted reproductive technology in the presence of polycystic ovary syndrome: current evidence and knowledge gaps. Endocr Pract. (2024) 30(1):64–9. 10.1016/j.eprac.2023.09.004 37708997

[11] Zhu X Fu Y. Extending letrozole treatment duration is effective in inducing ovulation in women with polycystic ovary syndrome and letrozole resistance. Fertil Steril. (2023) 119(1):107–13. 10.1016/j.fertnstert.2022.09.018 36283866

[12] Ratna MB Bhattacharya S McLernon DJ. External validation of models for predicting cumulative live birth over multiple complete cycles of IVF treatment. Hum Reprod. (2023) 38(10):1998–2010. 10.1093/humrep/dead165 37632223 PMC10546080

[13] Zhu S Chen X Li R Jiang W Zheng B Sun Y. Constructing a predictive model for live birth following fresh embryo transfer in antagonist protocol for polycystic ovary syndrome. J Assist Reprod Genet. (2024) 41(10):2709–19. 10.1007/s10815-024-03232-4 39168929 PMC11534932

[14] Collins GS Dhiman P Ma J Schlussel MM Archer L Van Calster B. Evaluation of clinical prediction models (part 1): from development to external validation. Br Med J. (2024) 384:e074819. 10.1136/bmj-2023-074819 38191193 PMC10772854

[15] Liu Y Gao J Ge H Feng J Wang Y Wu X. Development and validation of a LASSO logistic regression based nomogram for predicting live births in women with polycystic ovary syndrome: a retrospective cohort study. Front Endocrinol. (2025) 16:1525823. 10.3389/fendo.2025.1525823 PMC12148842 40496554

[16] Wang X Tian P-Z Zhao Y-J Lu J Dong C-Y Zhang C-L. The association between female age and pregnancy outcomes in patients receiving first elective single embryo transfer cycle: a retrospective cohort study. Sci Rep. (2024) 14(1):19216. 10.1038/s41598-024-70249-1 39160203 PMC11333704

[17] Sebastian-Leon P Sanz FJ Molinaro P Pellicer A Diaz-Gimeno P. Advanced maternal age was associated with an annual decline in reproductive success despite use of donor oocytes: a retrospective study. Fertil Steril. (2025) 124(4):635–44. 10.1016/j.fertnstert.2025.05.150 40403911

[18] Karabulut S Kutlu P Korkmaz O Oria L. Impact of maternal age on the repairing capacity of oocytes on paternal DNA damage. Reprod Sci. (2025) 32(7):2397–403. 10.1007/s43032-025-01911-w 40504287 PMC12271266

[19] Caldwell AE Gorczyca AM Bradford AP Nicklas JM Montgomery RN Smyth H. Effectiveness of preconception weight loss interventions on fertility in women: a systematic review and meta-analysis. Fertil Steril. (2024) 122(2):326–40. 10.1016/j.fertnstert.2024.02.038 38408693 PMC11384273

[20] Fernandes A Gordon A Sweeting A. Pre-conception weight loss interventions in women with polycystic ovary syndrome and the effect on perinatal outcomes: a quantitative synthesis of surrogate outcomes. Diabetes Obes Metab. (2025) 27(12):7158–79. 10.1111/dom.70116 41035114 PMC12587233

