# Supplementary material for: Development and temporal validation of a prediction model for live birth in infertile women with polyendocrine metabolic ovarian syndrome: a retrospective cohort study
Source: Front Reprod Health. 2026 Jul 28;8:1891181. doi: 10.3389/frph.2026.1891181 (PMC13457464; doi:10.3389/frph.2026.1891181)
Supplement: Supplementary file 1 [file Supplementaryfile1.docx]

**Supplementary Table S1. Complete Logistic Regression Equation and Predictor Coding of the Final Prediction Model**

| **Predictor** | **Coding** | ***β*** | ***OR*** | ***95%CI*** |
| --- | --- | --- | --- | --- |
| Intercept | — | 6.292 | — | — |
| Age | per year | -0.092 | 0.91 | 0.85–0.98 |
| BMI | per kg/m² | -0.083 | 0.92 | 0.86–0.98 |
| Infertility duration | per year | -0.161 | 0.85 | 0.77–0.94 |
| Gravidity | per pregnancy | -0.099 | 0.91 | 0.73–1.12 |
| LH/FSH ratio | continuous | -0.337 | 0.71 | 0.55–0.91 |
| Tubal/pelvic factor | 0=No, 1=Yes | -0.25 | 0.78 | 0.48–1.26 |
| Uterine factor | 0=No, 1=Yes | -1.004 | 0.37 | 0.15–0.80 |

**Supplementary Table S2. Missing data for candidate predictors and outcomes**

| **Variable** | **Development cohort (n=389), missing n (%)** | **Temporal validation cohort (n=221), missing n (%)** |
| --- | --- | --- |
| Female age | 0 (0.0%) | 0 (0.0%) |
| BMI | 0 (0.0%) | 0 (0.0%) |
| Infertility duration | 18 (4.6%) | 8 (3.6%) |
| Type of infertility | 0 (0.0%) | 0 (0.0%) |
| Gravidity | 0 (0.0%) | 0 (0.0%) |
| Parity | 0 (0.0%) | 0 (0.0%) |
| Miscarriage history | 0 (0.0%) | 0 (0.0%) |
| Ectopic pregnancy history | 0 (0.0%) | 0 (0.0%) |
| FSH | 23 (5.9%) | 10 (4.5%) |
| LH | 13 (3.3%) | 6 (2.7%) |
| LH/FSH ratio | 23 (5.9%) | 10 (4.5%) |
| Estradiol (E2) | 9 (2.3%) | 6 (2.7%) |
| Progesterone (P) | 12 (3.1%) | 9 (4.1%) |
| Prolactin (PRL) | 37 (9.5%) | 16 (7.2%) |
| Testosterone (T) | 31 (8.0%) | 15 (6.8%) |
| AMH | 369 (94.9%) | 220 (99.5%) |
| Tubal/pelvic factors | 0 (0.0%) | 0 (0.0%) |
| Ovulatory dysfunction | 0 (0.0%) | 0 (0.0%) |
| Uterine factors | 0 (0.0%) | 0 (0.0%) |
| Male factors | 0 (0.0%) | 0 (0.0%) |
| Thyroid disease | 0 (0.0%) | 0 (0.0%) |
| Abnormal glucose metabolism | 0 (0.0%) | 0 (0.0%) |
| Live birth within 365 days | 0 (0.0%) | 0 (0.0%) |
| Outcome verification through 365 days | 0 (0.0%) | 0 (0.0%) |

**Supplementary Table S3. Restricted cubic spline assessment of nonlinearity for continuous predictors**

| **Variable** | **P for nonlinearity** |
| --- | --- |
| Age | 0.059 |
| BMI | 0.36 |
| Infertility duration | 0.983 |
| LH/FSH ratio | 0.944 |

**Supplementary Table S4. Sensitivity analysis comparing the full prediction model with a reduced model excluding gravidity and tubal/pelvic factors**

| **Model** | **Cohort** | **AUC** | **Brier score** |
| --- | --- | --- | --- |
| Full model | Development | **0.735** | **0.208** |
| Reduced model* | Development | **0.725** | **0.210** |
| Full model | Temporal validation | **0.738** | **0.205** |
| Reduced model* | Temporal validation | **0.731** | **0.209** |

**Note:** *The reduced model excluded gravidity and tubal/pelvic factors. The reduced model showed only a slight decrease in discrimination, with minimal change in Brier score, supporting retention of these clinically relevant predictors in the final prediction model.

**Supplementary Table S5. Complete-case datasets used for final model estimation and temporal validation.**

| **Cohort** | **Eligible analytic cohort** | **Missing infertility duration** | **Missing LH/FSH ratio** | **Missing ≥1 final predictor** | **Complete cases** | **Live-birth events** | **No live birth** |
| --- | --- | --- | --- | --- | --- | --- | --- |
| Development cohort | 389 | 18 | 23 | 40 | 349 | 158 | 191 |
| Temporal validation cohort | 221 | 8 | 10 | 18 | 203 | 91 | 112 |
| Overall | 610 | 26 | 33 | 58 | 552 | 249 | 303 |

Note: One woman in the development cohort had missing values for both infertility duration and LH/FSH ratio. No overlap was observed in the temporal validation cohort.

**Figure S1. Participant flow diagram for cohort formation and complete-case model analyses.**
